# Timeline Kinetics of Systemic and Airway Immune Mediator Storm for Comprehensive Analysis of Disease Outcome in Critically Ill COVID-19 Patients

**DOI:** 10.3389/fimmu.2022.903903

**Published:** 2022-06-03

**Authors:** Juan Jonathan Gonçalves, Camila Pacheco Silveira Martins da Mata, Alice Aparecida Lourenço, Ágata Lopes Ribeiro, Geovane Marques Ferreira, Thais Fernanda de Campos Fraga-Silva, Fernanda Mesquita de Souza, Vanessa Egídio Silveira Almeida, Iara Antunes Batista, Carolina D`Avila-Mesquita, Ariel E. S. Couto, Ligia C. B. Campos, Adriana Alves Oliveira Paim, Linziane Lopes Ferreira, Patrícia de Melo Oliveira, Lorena de Almeida Teixeira, Daisymara Priscila de Almeida Marques, Henrique Retes de Moraes, Samille Henriques Pereira, Joaquim Pedro Brito-de-Sousa, Ana Carolina Campi-Azevedo, Vanessa Peruhype-Magalhães, Márcio Sobreira Silva Araújo, Andréa Teixeira-Carvalho, Flávio Guimarães da Fonseca, Vânia Luiza Deperon Bonato, Christiane Becari, Denise Ferro, Mayra Gonçalves Menegueti, Amanda Alves Silva Mazzoni, Maria Auxiliadora-Martins, Jordana Grazziela Coelho-dos-Reis, Olindo Assis Martins-Filho

**Affiliations:** ^1^ Instituto René Rachou, Fundação Oswaldo Cruz, Belo Horizonte, Brazil; ^2^ Departamento de Microbiologia, Instituto de Ciências Biológicas, Universidade Federal de Minas Gerais, Belo Horizonte, Brazil; ^3^ Hospital Risoleta Tolentino Neves, Universidade Federal de Minas Gerais, Belo Horizonte, Brazil; ^4^ Departamento de Bioquímica e Imunologia, Faculdade de Medicina de Ribeirão Preto, Universidade de São Paulo, Ribeirão Preto, Brazil; ^5^ Divisão de Cirurgia Vascular, Departamento de Cirurgia e Anatomia, Faculdade de Medicina de Ribeirão Preto, Universidade de São Paulo, Ribeirão Preto, Brazil; ^6^ Centro de Tecnologia em Vacinas da Universidade Federal de Minas Gerais (UFMG), Universidade Federal de Minas Gerais, Belo Horizonte, Brazil; ^7^ Escola de Enfermagem de Ribeirão Preto, Universidade de São Paulo, Ribeirão Preto, Brazil; ^8^ Divisão de Medicina Intensiva, Departamento de Cirurgia e Anatomia, Faculdade de Medicina de Ribeirão Preto, Universidade de São Paulo, Ribeirão Preto, Brazil

**Keywords:** COVID, cytokines, chemokines, growth factors, prognostic biomarkers, systemic, airway, immune mediators

## Abstract

In the present study, the levels of serum and airway soluble chemokines, pro-inflammatory/regulatory cytokines, and growth factors were quantified in critically ill COVID-19 patients (total n=286) at distinct time points (D0, D2-6, D7, D8-13 and D>14-36) upon Intensive Care Unit (ICU) admission. Augmented levels of soluble mediators were observed in serum from COVID-19 patients who progress to death. An opposite profile was observed in tracheal aspirate samples, indicating that systemic and airway microenvironment diverge in their inflammatory milieu. While a bimodal distribution was observed in the serum samples, a unimodal peak around D7 was found for most soluble mediators in tracheal aspirate samples. Systems biology tools further demonstrated that COVID-19 display distinct eccentric soluble mediator networks as compared to controls, with opposite profiles in serum and tracheal aspirates. Regardless the systemic-compartmentalized microenvironment, networks from patients progressing to death were linked to a pro-inflammatory/growth factor-rich, highly integrated center. Conversely, patients evolving to discharge exhibited networks of weak central architecture, with lower number of neighborhood connections and clusters of pro-inflammatory and regulatory cytokines. All in all, this investigation with robust sample size landed a comprehensive snapshot of the systemic and local divergencies composed of distinct immune responses driven by SARS-CoV-2 early on severe COVID-19.

## Introduction

In 2019, a mysterious respiratory disease required attention from the public health agencies in Hubei, China (1, 2). The disease was later named COVID-19, which was associated with the presence of the newly discovered RNA virus, the *severe acute respiratory syndrome-related coronavirus*, the SARS-CoV-2 (3). So far, it is estimated that around 435 million people were infected worldwide and about 6 million deaths occurred in less than two years of viral circulation (4). COVID-19 pandemic progressed fast and remarkably uncontrolled in contrast with another critical emerging coronavirus such as MERS and SARS-CoV-1.

COVID-19 is characterized by general symptoms such as fever, anosmia, tiredness, and dry cough. Other less common symptoms that may affect some patients are nasal congestion, conjunctivitis, sore throat, headache, muscle or joint pain, cutaneous rash, nausea or vomiting, diarrhea, chills, or dizziness (5). While most individuals infected with SARS-CoV-2 will develop mild symptoms, a significant percentage of patients may evolve to the severe form of the disease, which will demand mechanic ventilation and eventually lead to death (1, 2, 5, 6). In Brazil, the death rate amongst ICU admitted COVID-19 patients may vary according to region and hospitals, which ranged from 31.6% in March 2020 to 36.5% in March 2022 (7).

In this regard, it is still unclear why some critically ill COVID-19 patients survive, while others succumb to death. Studies have reported that individual risk of death might be influenced by host genetic factors. Genetic variants in genes related to cilia dysfunctions, cardiovascular and thromboembolic disease, mitochondrial impairment, and innate immune response have been identified (8). COVID-19 is featured by an augmented acute inflammatory response in which mediators such as C Reactive Protein, D-dimer, and troponin are abundant in serum samples from patients (6, 9). A conspicuous increase in inflammatory cytokines and chemokines is observed during infection, however, no evidence of their coordinated role on the fight against the virus is clearly demonstrated (10, 11). On the contrary, their increase can cause a deranged cytokine storm syndrome that contributes to hyper inflammation, failure in multiple organs and pathogenesis. Unraveling the features, timing and magnitude of cytokine storm would aid on design of the tailor-made therapeutics against the cytokine-mediated inflammatory injury during COVID-19. In this regard, therapeutic approaches under investigation are targeting the overactive cytokine release with anti-cytokine antibodies or immunomodulators, nonetheless a balanced maintenance and adequate inflammatory response for pathogen clearance must be considered, while abrogating immune molecules (12).

In a comprehensive evaluation of bronchoalveolar lavage from patients with moderate and severe seasonal Influenza and severe COVID-19 has revealed that the pulmonary compartment is composed of a robust overproduction of immediate proinflammatory response with the presence of tumor necrosis factor IL-6, IP-10, MCP-1, and CXCL8, TNF and IL-1β (13). In addition, COVID-19 patients have demonstrated an early and persistent transcriptional profile that indicates leukocyte activation in the severe form of the disease (14). These cytokines are associated to an increased risk of cardiovascular hyperpermeability, multiorgan failure, and eventually death when the high cytokine concentrations are unabated over time (15–21). It is also imperative to study the lungs and the upper airways in the context of SARS, which are of paramount importance for analyzing the resultant of the local molecular pattern in the respiratory milieu, essential for understanding the dynamics of immune activation/inflammation versus regulation during COVID-19 (10, 13, 18, 22, 23). Therefore, in the present study, several soluble mediators of serum and tracheal aspirate samples from a cohort of critically ill COVID-19 patients was evaluated in a longitudinal fashion. A divergent kinetics profile was observed in serum and tracheal aspirate samples from COVID-19, and a highly divergent pattern in serum and the respiratory secretion was also observed. These results bring insights in terms of timing and pattern of immunologic responses locally and systemically during COVID-19.

## Materials and Methods

### Study Population

This observational cross-sectional investigation was comprised of a convenience sample including 675 biological specimens (serum and tracheal aspirates) from critically ill COVID-19 patients admitted at intensive care unit (ICU) and controls at the peak of SARS-CoV-2 B1 lineage epidemiological status of hospital areas. No COVID-19 vaccination was available at the time of sample collection; therefore, all patients were unvaccinated. The main goal of this study was to analyze the systemic and airway compartmentalized immune response. The COVID-19 patients were enrolled at admission in the ICU from Hospital Risoleta Tolentino Neves (Belo Horizonte, MG) and from Hospital das Clínicas da Faculdade de Medicina de Ribeirão Preto da Universidade de São Paulo (Ribeirão Preto, SP). The COVID-19 diagnosis was confirmed by positive RT-PCR for SARS-CoV-2 targeting the E gene. A detailed compendium of study population and methods is presented in **Supplementary Figure 1**.

For assessing the systemic immune response, the “COVID” group comprised 477 serum samples from 183 COVID-19 patients (72 females and 111 males – age range of 18-90 years-old, median age of 65 years-old), obtained at five consecutive time points (Days = D), including: D0 (n=183), D2-6 (n=99), D7 (n=80), D8-13 (n=50) and D>14-36 (n=65) after ICU admission. The “COVID” group was further classified according to the disease outcome and referred as: “Discharge” (n=97, age average 58 years old) or “Death” (n=86, age average 65 years old). Serum samples were collected by venipuncture in the early morning routine of ICU visit, aliquoted and stored at -80°CC until processing. The control group was composed of 135 serum samples from age-matched pre-pandemic healthy controls referred as: “HC” group (70 females and 65 males – age range of 18-90 years-old, median age 65 years-old). Serum samples were obtained from a biorepository maintained at -80°CC at Instituto René Rachou (FIOCRUZ-Minas).

For assessing the airway compartmentalized immune response, the “COVID” group was composed of 198 tracheal aspirate (TA) samples from 103 COVID-19 patients (39 females and 64 males – age range of 18-90 years-old, median age 64 years-old), collected at five consecutive time points (Days = D), including: D0 (n=103), D2-6 (n=37), D7 (n=19), D8-13 (n=20) and D>14-36 (n=19) after ICU admission. The “COVID” group was further categorized according to the disease outcome, referred as: “Discharge” (n=37, age average 58 years old) or “Death” (n=66, age average 65 years old). The control group was composed of 18 non-infected patients (NI) under mechanic ventilation (5 females and 13 males – age range of 18-90 years-old, median age 53 years-old) due to trauma or burning, all presenting negative diagnosis of SARS-CoV-2 infection by RT-PCR. The sample collection for this group of control patients was performed at ICU admission just after intubation, therefore close to the day of injury (D0-D1). TA samples (2-5mL) were collected by aspiration into sterile tracheal secretion collectors in the early morning routine visit of patients for airway cleansing. Only productive secretion was included in this study. TA samples were immediately transferred to a biosecurity level 3 laboratory, aliquoted and stored at -80°CC until processing.

Baseline clinical data of critically ill COVID-19 patients was obtained from medical records. The most frequent comorbidities included: high body mass index – BMI>30 (58%), hypertension (57%) and diabetes (46%). The average of hospitalization was of 16 ± 2 days for COVID-19 patient group, being 18 ± 3 days for patients evolving to discharge and 14 ± 2 days for patients progressing to death. No significant differences were observed for comparative analysis of hospitalization days between discharge *vs* death. All patients have received corticosteroid treatment (Methylprednisolone 1mg/kg), prophylactic Enoxaparin (40U administered subcutaneous, once daily), and empirical antibiotic therapy, adjusted according to hemoculture results. No patient received antiviral therapy during ICU stay.

The study protocol was submitted and approved by the Ethical Committee of Instituto René Rachou/FIOCRUZ-Minas (CAAE: 42560721.7.0000.5091) and Hospital das Clínicas da Faculdade de Medicina de Ribeirão Preto - Universidade de São Paulo/USP-RP (CAAE: 30816620.0.0000.5440). This investigation followed the principles of Helsinki declaration as well as the resolution #466/2012 from the Brazilian Ministry of Health for research involving humans. Informed consent was obtained from all participants or their next of kin prior to inclusion in the present investigation.

### Serum and Tracheal Aspirate Preparation

Serum sample aliquots were thawed at 37°CC, centrifuged at 24,000 x *g* to remove lipid layer and large debris and the supernatant filtered through 0.45μm syringe filter to further maximize the debris removal. Tracheal aspirate (TA) aliquots were thawed at 37°CC, diluted in 1:2 in phosphate buffered saline (PBS) filtered through cell strainers (70µm) into polypropylene tubes to remove mucous clumps and cell aggregates. Samples were further centrifuged at 24,000 x *g* to remove and large debris and the supernatant filtered through 0.45μm syringe filter to further maximize the debris removal. Pre-filtered serum and TA samples were maintained on ice until use on the high-throughput microbeads array.

### Assessment of Soluble Immune Mediators in Serum and Tracheal Aspirate Samples

The levels of soluble immune mediators were measured in serum samples from COVID-19 patients (COVID), age-matched pre-pandemic healthy controls (HC) as well as in tracheal aspirate samples from COVID-19 patients (COVID) and non-infected patients (NI) by high-throughput microbeads array (Bio-Plex Pro™ Human Cytokine 27-plex Assay, Bio-Rad Laboratories, Hercules, CA, USA), following the manufacturer’s instructions. A range of immune mediators were quantified, including: chemokines (CXCL8, CCL11, CCL3, CCL5, CCL4, CCL2, and CXCL10), proinflammatory cytokines (IL-1β, IL-6, TNF, IL-12, IFN-γ, IL-15 and IL-17), regulatory cytokines (IL-1Ra, IL-4, IL-5, IL-9, IL-10, IL-13) and growth factors (FGF-basic, PDGF, VEGF, G-CSF, GM-CSF, IL-7 and IL-2). The results were expressed in pg/mL according to standard curves for each immune mediator using a fifth parameter logistic fit analysis. Luminex analysis were carried out in a biosecurity level 3 laboratory facility at Federal University of Minas Gerais and data acquisition performed at the Flow Cytometry core facility at FIOCRUZ-MINAS, using the Bio-plex 200 System (Bio Rad Laboratories, CA, USA).

### Data Mining and Statistical Analysis

All statistical analysis were carried out using GraphPad Prism, version 8.0, (San Diego, CA, USA). Comparative analysis between two independent groups were carried out by Student t-test for parametric data and by Mann-Whitney test for non-parametric data. For multiple comparisons amongst subgroups, all data that followed parametric distribution were analyzed by one-way ANOVA followed by Tukey’s *post-hoc* test. For non-parametric data, Kruskal-Wallis test was applied, followed by the Dunn’s multiple comparisons test amongst subgroups. The profile of serum and TA soluble immune mediators along the kinetic timeline was performed by a cross-sectional analysis along with five consecutive scheduled time points (Days = D), including D0-1, D2-6, D7, D8-13, D>14-36 after ICU admission by Kruskal-Wallis test followed by Dunn’s post-test for pairwise comparisons with the immediately earlier time-point. Additionally, the Mann-Whitney test was employed for comparisons between subgroups at matching time-points. In all cases, a threshold of p<0.05 was considered for statistical significance.

For correlation analysis of data with parametric distribution, Pearson’s correlation test was employed, whereas Spearman correlation test was employed for data with non-parametric distribution. The significant “r” scores were employed to assemble comprehensive network matrices.

Networks were built using a layout based on eccentricity employing the Cytoscape open-source software platform for visualizing complex networks (available at https://cytoscape.org). The network nodes represent the serum and TA soluble immune mediators, including chemokines, pro-inflammatory cytokines, regulatory cytokines and growth factors. The connecting edges illustrate weak/moderate (“r” scores between |0.1 to 0.67|) and strong correlations (“r” scores ≥ |0.67|) between pairs of attributes. Attributes without strong correlations are distributed in the network periphery. Serum soluble mediators presenting at least 5 strong correlations are assembled in the center with the number of serum soluble mediators and connections between them used to define the central connectivity.

The analysis of magnitude of changes in the serum and TA soluble immune mediators in COVID-19 patients were performed by calculating the proportion ratio between the individual values for each soluble immune mediator over the median value observed in the respective serum (HC) and TA (NI) control groups.

## Results

### Overall Snapshot of Serum Soluble Immune Mediators in Critically Ill COVID-19 Patients

**Figure 1:**
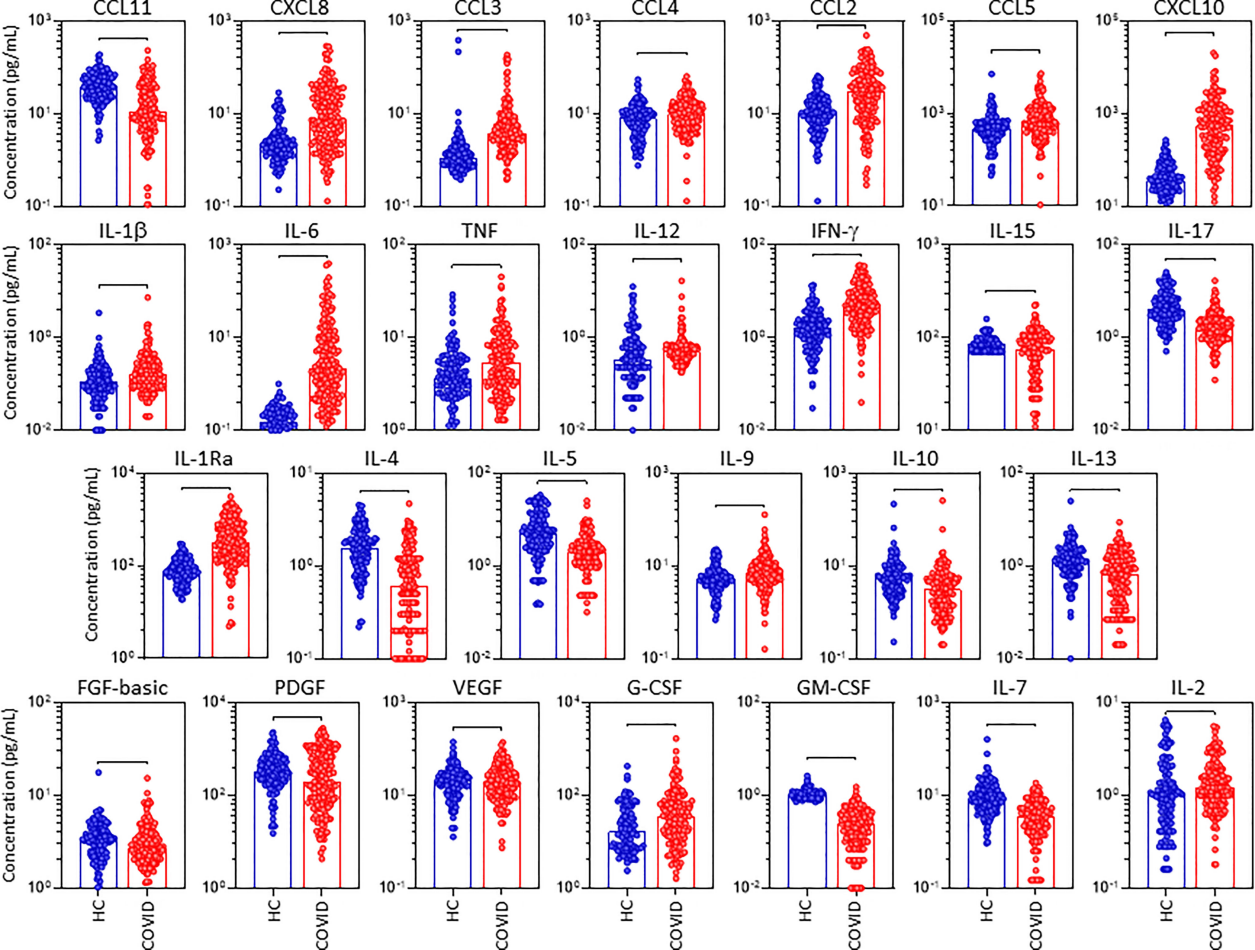
Overall snapshot of serum soluble immune mediators in critically ill COVID-19 patients at ICU admission. The concentration of chemokines (CXCL8, CCL11, CCL3, CCL4, CCL2, CCL5, CXCL10), pro-inflammatory cytokines (IL-1β, IL-6, TNF, IL-12, IFN-γ, IL-15, IL-17), regulatory cytokines (IL-1Ra, IL-4, IL-5, IL-9, IL-10, IL-13) and growth factors (FGF-basic, PDGF, VEGF, G-CSF, GM-CSF, IL-7 and IL-2) were quantified in serum samples from critically ill COVID-19 patients – “COVID” (●, n = 183), as compared to pre-pandemic healthy controls – “HC” (●, n = 135) at ICU admission (D0=baseline). Measurements were carried by high-throughput microbeads array as described in Material and Methods section. The results are expressed in pg/mL and presented as scattering distribution of individual values over bar plots underscoring the median value on each bar. Significant differences at p <0.05 are indicated by connecting lines.

In order to characterize in detail, the systemic immune profile of severe COVID-19, the serum concentration of chemokines, pro-inflammatory/regulatory cytokines and growth factors were quantified in serum samples from critically ill COVID-19 patients (COVID) as compared to pre-pandemic healthy controls (HC) (**Figure 1**). Data analysis demonstrate COVID group presented an upregulation of several serum soluble immune mediators, featured by the increased levels of CXCL8, CCL3, CCL4, CCL2, CXCL10, IL-1β, IL-6, TNF, IL-12, IFN-γ, IL-1Ra, IL-9, G-CSF, and IL-2 along with decreased levels of CCL11, CCL5, IL-15, IL-17, IL-4, IL-5, IL-10, IL-13, FGF-basic, PDGF, VEGF, GM-CSF and IL-7 (**Figure 1**). The magnitude of changes in serum soluble immune mediators are shown in the **Supplementary Figure 2**. The results demonstrated that CXCL10(15x), IL-6(14x), IL-1Ra;CXCL8(4x) and CCL3;IFN-γ;CCL2(3x) presented the highest increments according to the median values observed in the pre-pandemic healthy controls. On the other hand, IL-17;IL-5;IL-4;IL-7 (-0.4x), CCL11(-0.3x) and GM-CSF(-0.2x) displayed the lowest values according to the median values observed for the HC group (**Supplementary Figure 2**).

### Panoramic Overview of Serum Soluble Immune Mediators in Critically Ill COVID-19 Patients According to Disease Outcome

**Figure 2:**
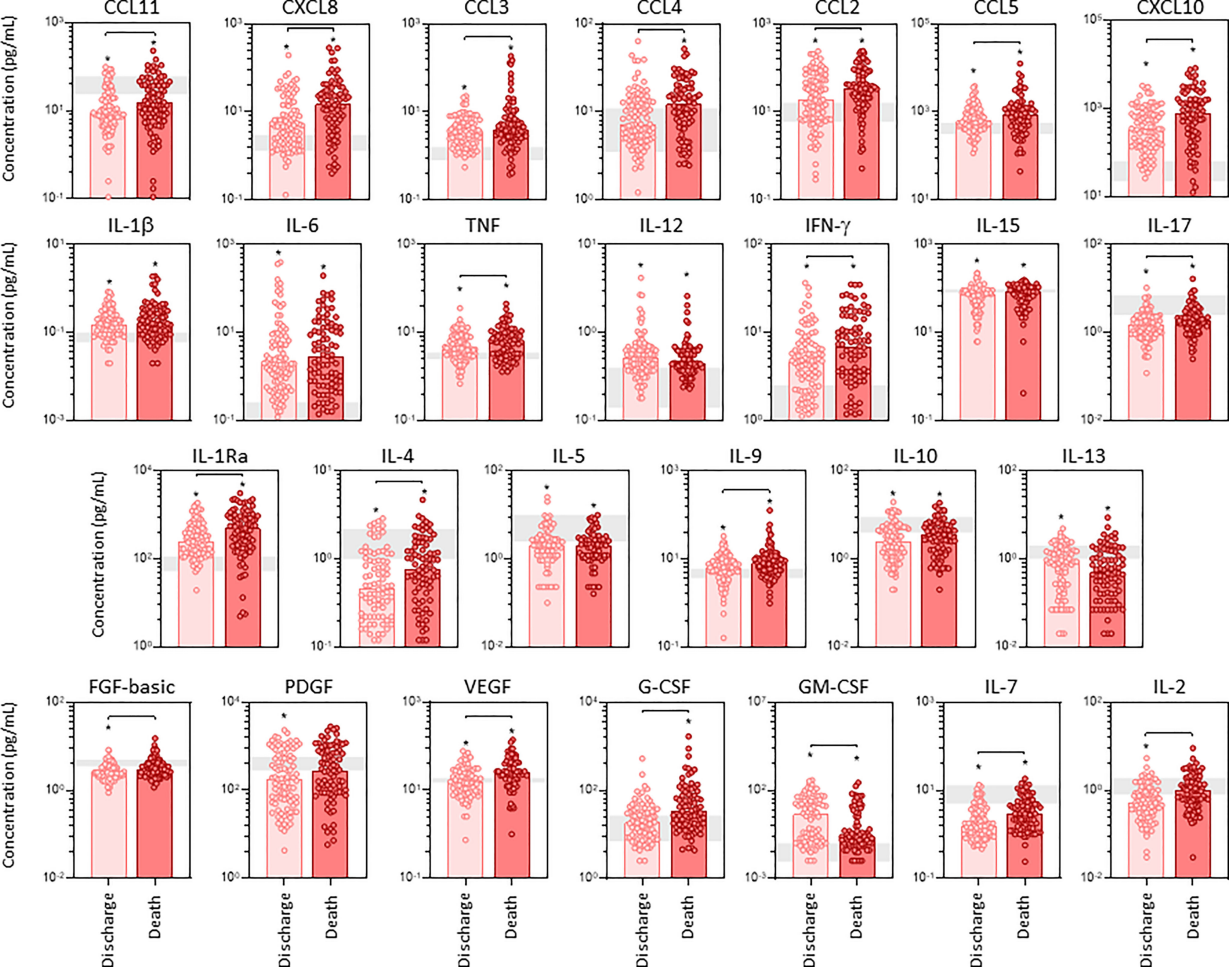
Panoramic overview of serum soluble immune mediators in critically ill COVID-19 patients at ICU admission, according to disease outcome. The concentration of chemokines (CXCL8, CCL11, CCL3, CCL4, CCL2, CCL5, CXCL10), pro-inflammatory cytokines (IL-1β, IL-6, TNF, IL-12, IFN-γ, IL-15, IL-17), regulatory cytokines (IL-1Ra, IL-4, IL-5, IL-9, IL-10, IL-13) and growth factors (FGF-basic, PDGF, VEGF, G-CSF, GM-CSF, IL-7 and IL-2) were quantified in serum samples from critically ill COVID-19 patients – “COVID” (n = 183) at ICU admission (D0=baseline), further categorized according to disease outcome, referred as “Discharge” (●, n = 97) or “Death” (●, n = 86). The gray zone represents the reference interquartile range (25^th^-75^th^) observed for pre-pandemic healthy controls “HC” (n = 135). Measurements were carried by high-throughput microbeads array as described in Material and Methods section. The results are expressed in pg/mL and presented as scattering distribution of individual values over bar plots underscoring the median value on each bar. Significant differences at p < 0.05 are indicated by asterisk (*) as compared to HC and by connecting lines for comparisons between Discharge versus Death.

The profile of serum soluble mediators in critically ill COVID-19 patients was further assessed according to the disease outcome: discharge or death (**Figure 2**). The results showed that increased levels of chemokines (CXCL8, CCL11, CCL3, CCL4, CCL2, CCL5, CXCL10), pro-inflammatory cytokines (TNF, IFN-γ, IL-17), regulatory cytokines (IL-1Ra, IL-4, IL-9) and growth factors (FGF-basic, VEGF, G-CSF, IL-7 and IL-2) were observed in COVID patients evolving to death as compared to the discharge subgroup. Conversely, the levels of GM-CSF were decreased in COVID patients progressing to death as compared to discharge (**Figure 2**). Further analysis of magnitude of changes in serum soluble immune mediators demonstrate that CXCL10 (21x), IL-6(20x), CXCL8;IL-1Ra(7x), IFN-γ(5x), CCL3(4x) and CCL2;G-CSF(3x) achieved the highest up-regulation in COVID patients evolving to death while IL-6(13x), CXCL10 (9x) and IL-1Ra;CCL3;IFN-γ(3x) were increased in lesser magnitude in patients progressing to discharge (**Supplementary Figure 2**). A general decrease (<-0.4) of CCL11, IL-17, IL-5, GM-CSF and IL-7 were observed in COVID patients regardless the disease outcome, with IL-4(-0.3x) and IL-13(-0.2x) observed in patients evolving to discharge or death, respectively (**Supplementary Figure 2**).

### Integrative Correlation Analysis and Networks of Soluble Immune Mediators in Serum Samples From Critically Ill COVID-19 Patients

**Figure 3:**
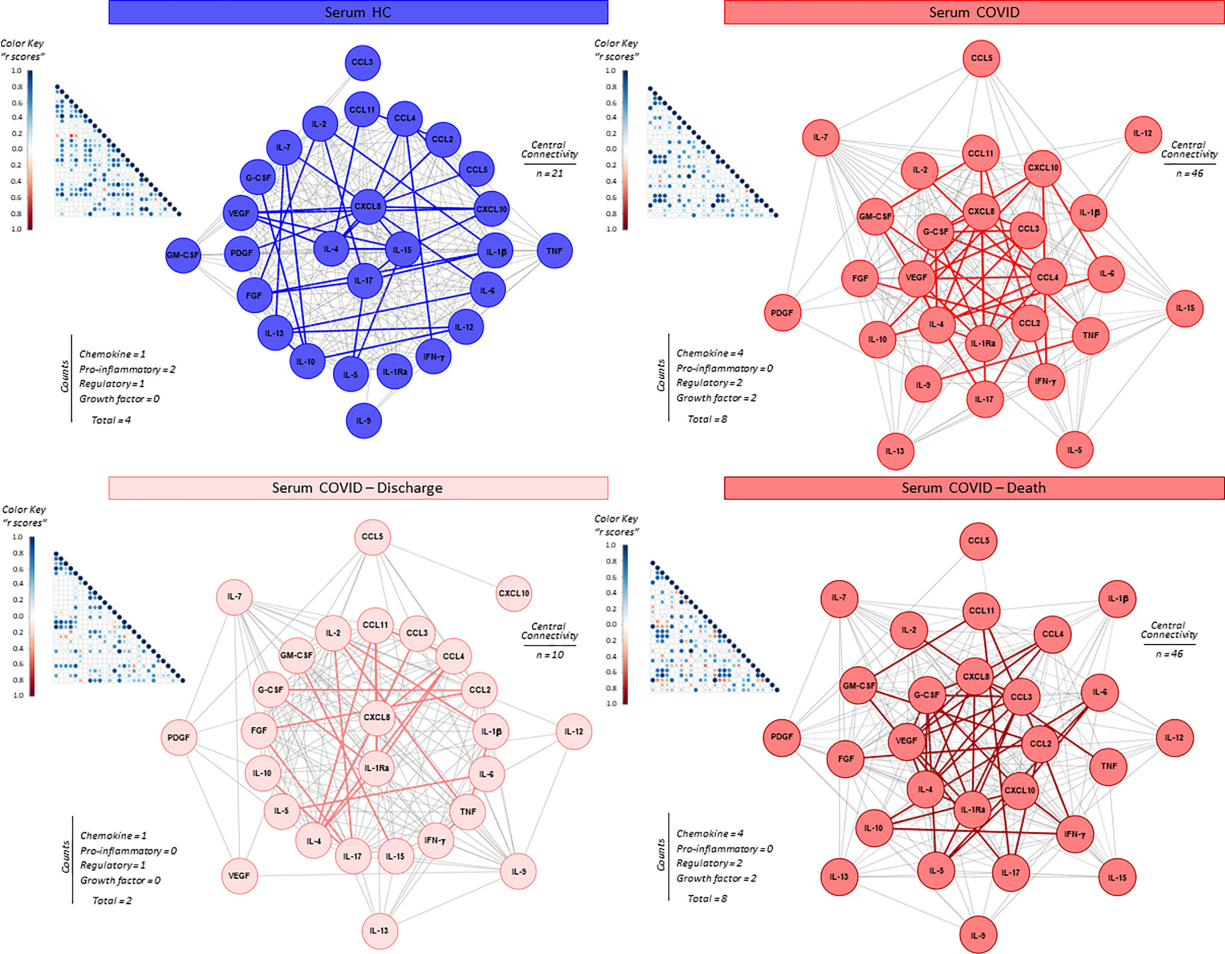
Integrative correlation matrices and networks analysis of serum soluble immune mediators in critically ill COVID-19 patients at ICU admission. Comprehensive correlation matrices were assembled based on the Pearson and Spearman “r” scores between chemokines, pro-inflammatory cytokines, regulatory cytokines and growth factors measured in serum samples from critically ill COVID-19 patients – “COVID” (●, n = 183) at ICU admission (D0=baseline), further classified according to disease outcome: referred as “Discharge” (●, n = 97) or “Death” (●, n = 86) and pre-pandemic healthy controls – “HC” (●, n = 135). The soluble mediators were measured by high-throughput microbeads array as described in Material and Methods section. Panoramic correlation matrices are shown as triangle templates with each square intersection representing the “r” correlation score between pairs of attributes. The “r” scores of significant correlations (p < 0.05) are represented by circles of proportional sizes, scaled from −1 to +1 with gradient for negative (red dots) and positive (bule dots) according to the color key provided in the figure. The white squares represent non-significant correlations. Networks were built using a layout based on eccentricity, considering all significant correlations. The nodes represent the chemokines (CXCL8, CCL11, CCL3, CCL4, CCL2, CXCL10), pro-inflammatory cytokines (IL-1β, IL-6, TNF, IL-12, IFN-γ, IL-15, IL-17), regulatory cytokines (IL-1Ra, IL-4, IL-5, IL-9, IL-10, IL-13) and growth factors (FGF-basic, PDGF, VEGF, G-CSF, GM-CSF, IL-7 and IL-2). Connecting edges illustrate weak/moderate (“r” scores between |0.1 to 0.67|, thin gray lines) and strong correlation (“r” scores ≥ |0.67|, thick colored lines) between pairs of attributes. Negative correlations are underscored by dashed lines. Attributes without strong correlations are distributed in the network periphery. Serum soluble mediators presenting at least 5 strong correlations are assembled in the center with the number of each category and connections between them (central connectivity) are provided in the Figure.

The interaction amongst serum soluble mediators in critically ill COVID-19 patients was assessed by system biology approaches based on comprehensive correlation matrices and networks and the data presented in the **Figure 3**. The results showed that COVID patients exhibited a network shows with higher entropic degree with higher number of central connectivity (n=46), featured by a strong center of 8 soluble immune mediators (CXCL8, CCL3, CCL4, CCL2, IL-1Ra, IL-4, VEGF and G-CSF). Conversely, the HC displayed a network with a small central cluster formed by only 4 molecules (CXCL8, IL-15, IL-17 and IL-4) (**Figure 3**).

The network profile of COVID patients progressing to death revealed a higher entropic pattern, with strong and well-knit correlation center (CXCL8, CCL3, CCL2, CXCL10, IL-1Ra, IL-4, VEGF and G-CSF), contrasting to that observed for COVID patients evolving to discharge (CXCL8 and IL-1Ra) (**Figure 3**).

### Kinetic Timeline of Serum Soluble Immune Mediators in Critically Ill COVID-19 Patients

**Figure 4:**
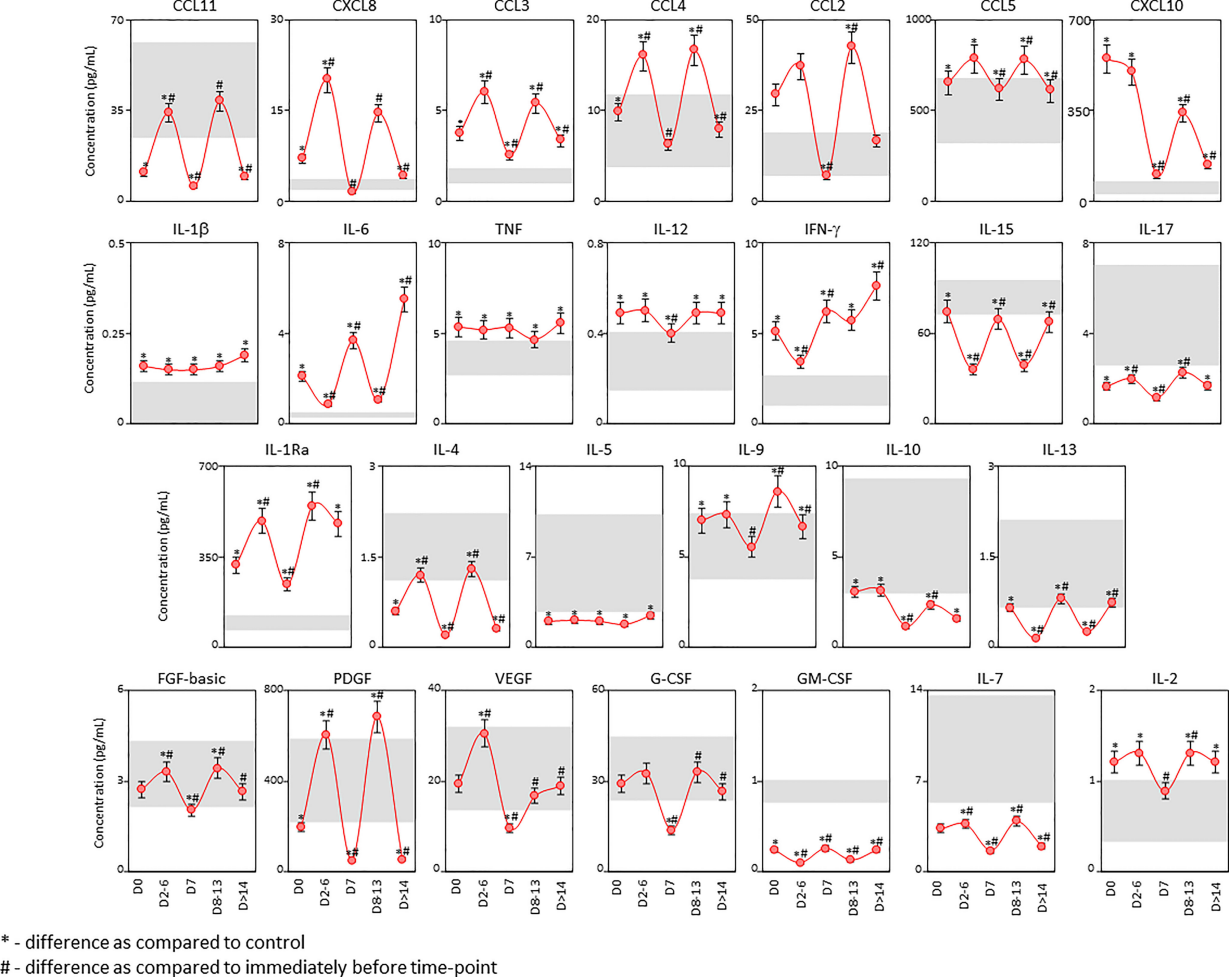
Kinetic timeline of soluble immune mediators in serum samples from critically ill COVID-19 patients. The kinetic profile of chemokines (CXCL8, CCL11, CCL3, CCL4, CCL2, CCL5, CXCL10), pro-inflammatory cytokines (IL-1β, IL-6, TNF, IL-12, IFN-γ, IL-15, IL-17), regulatory cytokines (IL-1Ra, IL-4, IL-5, IL-9, IL-10, IL-13) and growth factors (FGF-basic, PDGF, VEGF, G-CSF, GM-CSF, IL-7 and IL-2) was evaluated in serum samples from critically ill COVID-19 patients (●, n = 183) by cross-sectional analysis at four consecutive time points (Days = D), including: D0 (n = 183), D2-6 (n = 99), D7 (n = 80), D8-13 (n = 50) and D>14-36 (n = 65) after ICU admission. Measurements were carried out by high-throughput microbeads array as described in Material and Methods section. The results are presented as a line chart of median values (± 10% of median) at each time point along the kinetic timeline. The gray zone represents the reference interquartile range (25^th^-75^th^) observed for pre-pandemic healthy controls “HC” (n = 135). Significant differences at p < 0.05 are identified by asterisks (*) as compared with “HC” and by hashtag (#) for immediately preceding time-point.

Intending to further investigate the temporal profile of serum soluble mediators in critically ill COVID-19 patients, a cross-sectional kinetic timeline analysis was carried out at four consecutive time points (Days = D), including: D0 (n=183), D2-6 (n=99), D7 (n=80), D8-13 (n=50) and D>14-36 (n=65) after ICU admission (**Figure 4**). Data analysis demonstrated that a clear trend for a bimodal kinetics wave of serum soluble immune mediators in COVID patients, except for IL-1β, TNF and IL-5. Peaks at D2-6 and D8-13 were observed for chemokines (CXCL8, CCL11, CCL3, CCL4, CCL2, CCL5, CXCL10), pro-inflammatory cytokines (IL-12, IL-17), regulatory cytokines (IL-1Ra, IL-4, IL-9, IL-10) and growth factors (FGF-basic, PDGF, G-CSF, IL-7, and IL-2). Conversely, inverted bimodal profile was observed for IL-6, IFN-γ, IL-15, IL-13 and GM-CSF with valleys observed in the kinetics timeline at D2-6 and D8-13 (**Figure 4**).

The kinetic profile of critically ill COVID-19 patients classified according to the disease outcome of discharge or death were also evaluated (**Supplementary Figure 3**). The results demonstrated that patients progressing to death displayed a time-matching increase in almost all soluble immune mediators as compared to patients evolving to discharge, except for IL-12, IL-5, and IL-13. Of note, the levels of PDGF were significantly lower at D2-6 in patients progressing to death as compared to patients evolving to discharge. Nonetheless, CCL3, IL-15, IL-17, PDGF, VEGF and GM-CSF only presented differences in earlier time points up to D7 (**Supplementary Figure 3**).

### Landscape of Soluble Immune Mediators in Tracheal Aspirate Samples From Critically Ill COVID-19 Patients

**Figure 5:**
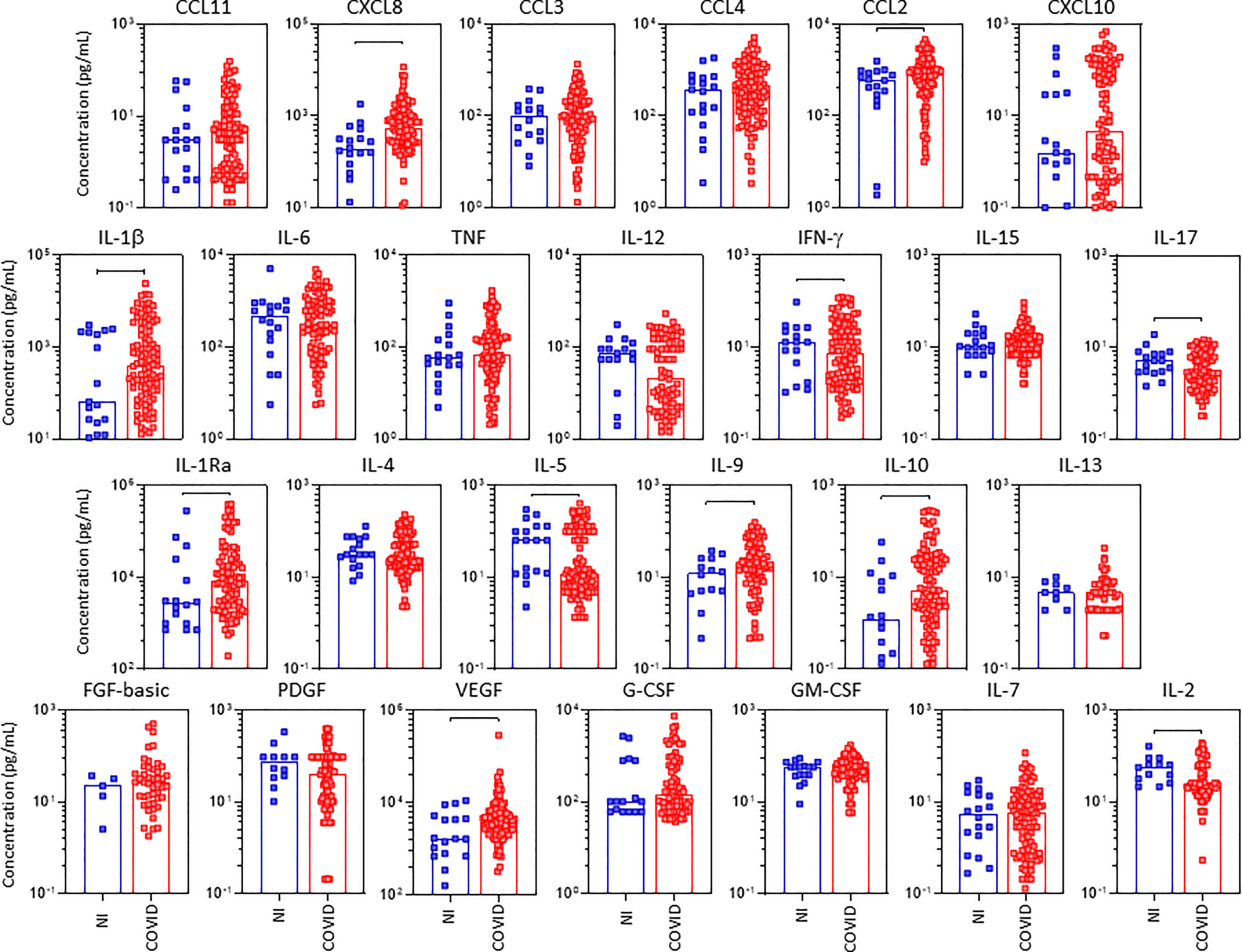
Landscape of airway soluble immune mediators in tracheal aspirate samples from critically ill COVID-19 patients at ICU admission. The concentration of chemokines (CXCL8, CCL11, CCL3, CCL4, CCL2, CXCL10), pro-inflammatory cytokines (IL-1β, IL-6, TNF, IL-12, IFN-γ, IL-15, IL-17), regulatory cytokines (IL-1Ra, IL-4, IL-5, IL-9, IL-10, IL-13) and growth factors (FGF-basic, PDGF, VEGF, G-CSF, GM-CSF, IL-7 and IL-2) were quantified in tracheal aspirate samples from critically ill COVID-19 patients – “COVID” (■, n = 103) at ICU admission (D0 = baseline), as compared to non-infected patients – “NI” (■, n = 18). Measurements were carried by high-throughput microbeads array as described in Material and Methods section. The results are expressed in pg/mL and presented as scattering distribution of individual values over bar plots underscoring the median value on each bar. Significant differences at p < 0.05 are indicated by connecting lines.

The concentration of chemokines, pro-inflammatory cytokines, regulatory cytokines and growth factors were quantified in tracheal aspirate samples from critically ill COVID-19 patients – “COVID”, as compared to non-infected patients – “NI” under mechanical ventilation at ICU (**Figure 5**). The results showed a more restricted and selective increase of CXCL8, CCL2, IL-1β, IL-1Ra, IL-9, IL-10, and VEGF as well as decreased levels of IFN-γ, IL-17, IL-5, and IL-2 as compared to NI (**Figure 5**). The magnitude of changes in tracheal aspirate soluble immune mediators are shown in the **Supplementary Figure 2**. The results demonstrated that IL-10;IL-1β(4x) and CXCL10;CXCL8;IL-1Ra(3x) exhibited the highest increments according to the median values observed in the NI control group. On the other hand, IL-12(-0.3x) and IL-5(-0.2x) showed the lowest values according to the median values observed for the NI group (**Supplementary Figure 2**).

### Profile of Soluble Immune Mediators in Tracheal Aspirate Samples From Critically Ill COVID-19 Patients According to Disease Outcome

**Figure 6:**
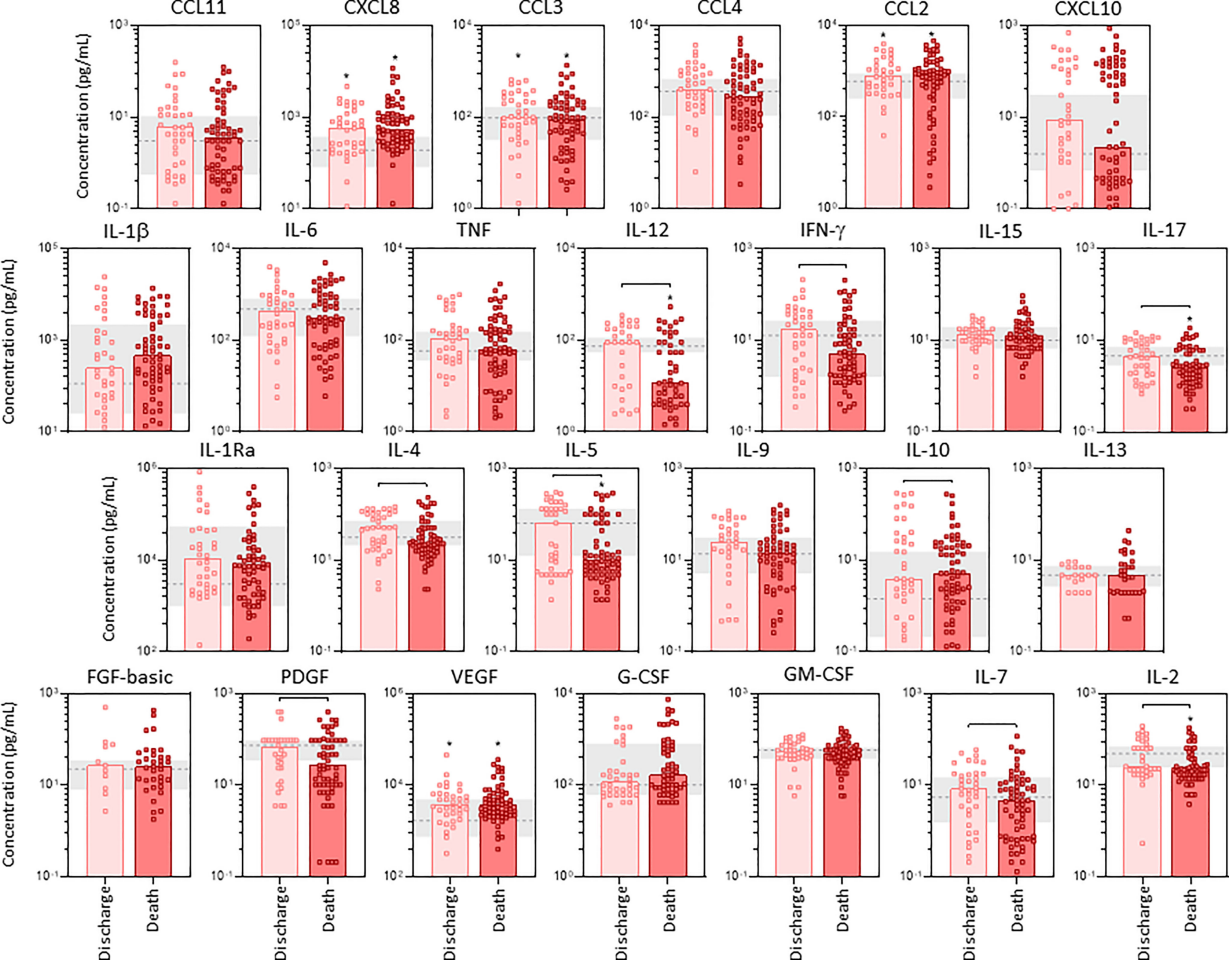
Profile of airway soluble immune mediators in tracheal aspirate samples from critically ill COVID-19 patients at ICU admission, according to disease outcome. The concentration of chemokines (CXCL8, CCL11, CCL3, CCL4, CCL2, CXCL10), pro-inflammatory cytokines (IL-1β, IL-6, TNF, IL-12, IFN-γ, IL-15, IL-17), regulatory cytokines (IL-1Ra, IL-4, IL-5, IL-9, IL-10, IL-13) and growth factors (FGF-basic, PDGF, VEGF, G-CSF, GM-CSF, IL-7 and IL-2) were quantified in tracheal aspirate samples from critically ill COVID-19 patients – “COVID” (n=103) at ICU admission (D0=baseline), further categorized according to disease outcome, referred as “Discharge” (■, n = 37) or “Death” (■, n = 66). The gray zone represents the reference interquartile range (25^th^-75^th^) observed for non-infected patients “NI” (n=18). Measurements were carried by high-throughput microbeads array as described in Material and Methods section. The results are expressed in pg/mL and presented as scattering distribution of individual values over bar plots underscoring the median value on each bar. Significant differences at p < 0.05 are indicated by asterisk (*) as compared to NI and by connecting lines for comparisons between Discharge versus Death.

The profile of serum soluble mediators in TA from critically ill COVID-19 patients was further characterized based on the disease outcome: discharge or death (**Figure 6**). Data analysis demonstrated that COVID patients progressing to death displayed significantly lower levels of pro-inflammatory cytokines IL-12, IFN-γ, IL-17, regulatory cytokines IL-4, IL-5 and growth factors PDGF, IL-7 and IL-2 as compared to the discharge subgroup. Conversely, the regulatory cytokine IL-10 was the sole molecule elevated in COVID patients evolving to death as compared to the discharge outcome (**Figure 6**). Further analysis of magnitude of changes in serum soluble immune mediators demonstrate that IL-10;IL1β(4x) and CXCL8;IL-1Ra(3x) achieved the highest up-regulation in COVID patients evolving to death while CXCL10(5x), IL-1Ra (4x) and CXCL8;IL-10(3x) were increased in patients progressing to discharge (**Supplementary Figure 2**). While a decrease (<-0.4) of IL-2, IFN-g, PDGF, IL-5 and IL-12 were observed in COVID patients progressing to death, no significant decrease was observed in COVID patients evolving to discharge (**Supplementary Figure 2**).

### Integrative Correlation Matrices and Networks Analysis of Soluble Immune Mediators in Tracheal Aspirates From Critically Ill COVID-19 Patients

**Figure 7:**
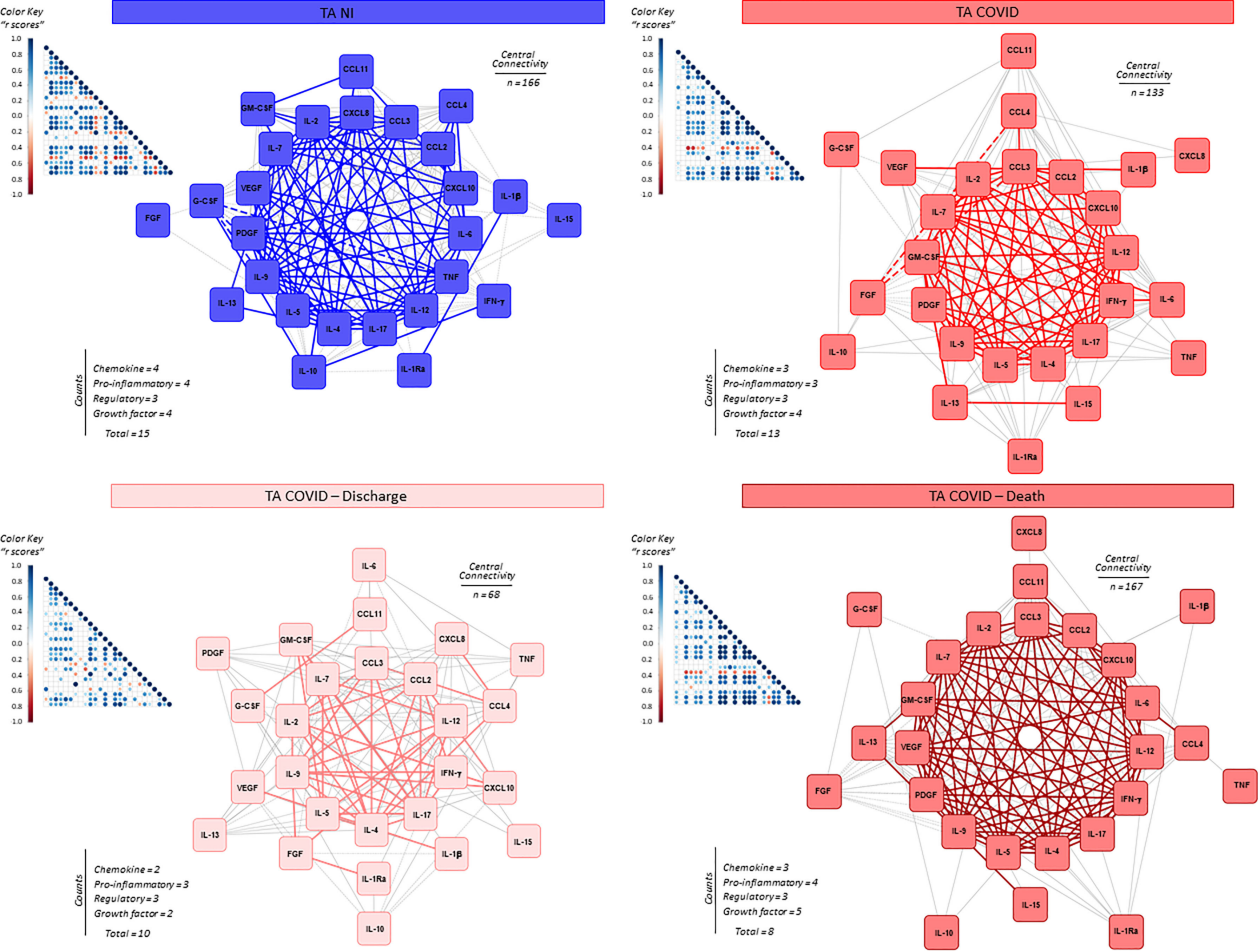
Integrative correlation matrices and networks analysis of airway soluble immune mediators in tracheal aspirates from critically ill COVID-19 patients at ICU admission. Comprehensive correlation matrices were assembled based on the Pearson and Spearman “r” scores between chemokines, pro-inflammatory cytokines, regulatory cytokines, and growth factors measured in tracheal aspirates from critically ill COVID-19 patients – “COVID” (■, n = 103) at ICU admission (D0=baseline), further classified according to disease outcome: referred as “Discharge” (■, n = 37) or “Death” (■, n = 66) and non-infected patients – “NI” (■, n = 18). The soluble mediators were measured by high-throughput microbeads array as described in Material and Methods section. Panoramic correlation matrices are shown as triangle templates with each square intersection representing the “r” correlation score between pairs of attributes. The “r” scores of significant correlations (p < 0.05) are represented by circles of proportional sizes, scaled from −1 to +1 with gradient for negative (red dots) and positive (bule dots) according to the color key provided in the figure. The white squares represent non-significant correlations. Networks were built using a layout based on eccentricity, considering all significant correlations. The nodes represent the chemokines (CXCL8, CCL11, CCL3, CCL4, CCL2, CXCL10), pro-inflammatory cytokines (IL-1β, IL-6, TNF, IL-12, IFN-γ, IL-15, IL-17), regulatory cytokines (IL-1Ra, IL-4, IL-5, IL-9, IL-10, IL-13) and growth factors (FGF-basic, PDGF, VEGF, G-CSF, GM-CSF, IL-7 and IL-2). Connecting edges illustrate weak/moderate (“r” scores between |0.1 to 0.67|, thin gray lines) and strong correlations (“r” scores ≥ |0.67|, thick colored lines) between pairs of attributes. Negative correlations are underscored by dashed lines. Attributes without strong correlations are distributed in the network periphery. Serum soluble mediators presenting at least 5 strong correlations are assembled in the center with the number of each category and connections between them (central connectivity) are provided in the Figure.

System biology approaches were employed to build comprehensive correlation matrices alongside eccentric networks to understand the integrative connectivity amongst soluble immune mediators in tracheal aspirates from critically ill COVID-19 patients (**Figure 7**). The results demonstrated that COVID patients displayed a loss of central connectivity as compared to NI. Moreover, COVID patients progressing to death presented higher number of connectivity between chemokines, proinflammatory cytokines and growth factors as compared to COVID patients evolving to discharge, which presented considerably lower number of connections without participation of growth factors (**Figure 7**).

### Kinetics of Soluble Immune Mediators in Tracheal Aspirate Samples From Critically Ill COVID-19 Patients

**Figure 8:**
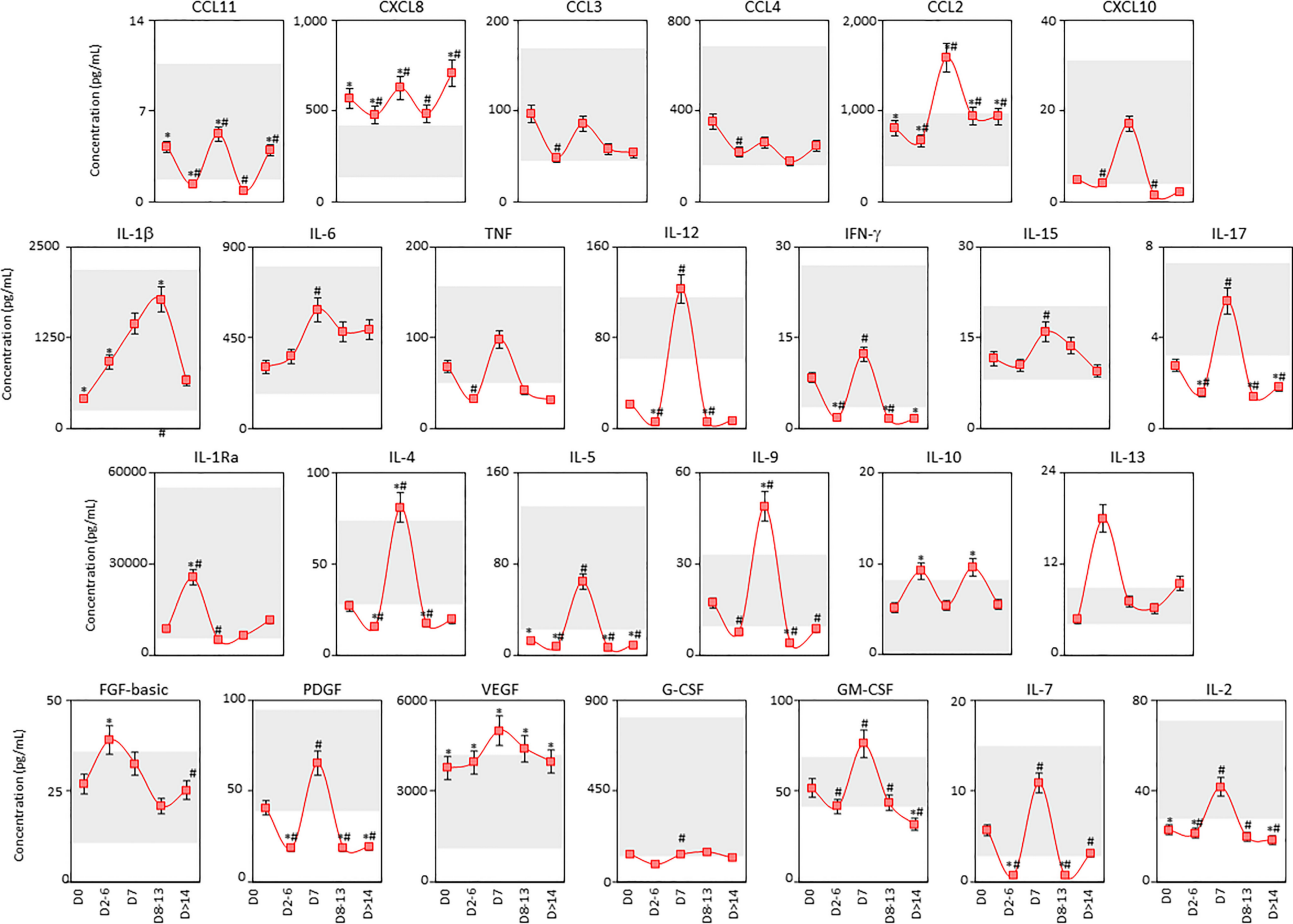
Kinetics of airway soluble immune mediators in tracheal aspirate samples from critically ill COVID-19 patients. The kinetic timeline of chemokines (CXCL8, CCL11, CCL3, CCL4, CCL2, CXCL10), pro-inflammatory cytokines (IL-1β, IL-6, TNF, IL-12, IFN-γ, IL-15, IL-17), regulatory cytokines (IL-1Ra, IL-4, IL-5, IL-9, IL-10, IL-13) and growth factors (FGF-basic, PDGF, VEGF, G-CSF, GM-CSF, IL-7 and IL-2) was evaluated in tracheal aspirate samples from critically ill COVID-19 patients (■, n = 103) by cross-sectional analysis at five consecutive time points (Days = D), including: D0 (n=103), D2-6 (n=37), D7 (n=19), D8-13 (n=20) and D > 14-36 (n=19) after ICU admission. Measurements were carried out by high-throughput microbeads array as described in Material and Methods section. The results are presented as a line chart of median values and interquatile range at each time point along the kinetic timeline. The gray zone represents the reference interquartile range (25^th^-75^th^) observed for non-infected patients “NI” (n=18). Significant differences at p < 0.05 are identified by asterisks (*) as compared with “NI” and by hashtag (#) for immediately preceding time-point.

The kinetic timeline of chemokines, pro-inflammatory cytokines, regulatory cytokines and growth factors was characterized in tracheal aspirate samples from critically ill COVID-19 patients by cross-sectional analysis at four consecutive time points studied after ICU admission (**Figure 8**). Data analysis showed that most soluble mediators presented an inverted bimodal distribution, with valleys at D2-6 and D8-13 and a peak around D7 (**Figure 8**). Interestingly, IL-10 presented an opposite bimodal distribution with peaks at D2-6 and D8-13 (**Figure 8**).

The analysis of TA soluble immune mediators was also performed in the light of the disease outcome to discharge or death (**Supplementary Figure 4**). Data analysis demonstrated that COVID patients progressing to death displayed lower levels of CXCL10, IL-6, IL-12, IFN-γ, IL-17, IL-4, IL-5, IL-10, PDGF, G-CSF, GM-CSF, IL-7 and IL-2 as compared to COVID patients evolving to discharge (**Supplementary Figure 4**).

## Discussion

The SARS-CoV-2 pandemic and the life-threatening COVID-19 have highlighted the crucial role of an effective/balanced host immune response and the destructive impact of immune dysregulation, that could lead not only severe but fatal outcome (11).

There has been an avid debate in terms whether severe COVID-19 is in fact a cytokine storm syndrome (10). However, it is well known that hyperinflammation is a key component of severe COVID-19, usually associated with poor outcomes. It has been proposed that the “cytokine storm” is a multifactorial event involving elevated levels of circulating immune mediators (10). It is unknown or there is no consensus of which cytokine is associated to protective versus pathogenic response.

However, the detailed mechanisms associated with this hyperactivation has not been completely characterized and differences in timing at systemic and airway microenvironment may represent a key event to understand the pathophysiology of COVID-19. Aiming at better characterizing this phenomenon, the present study was designed to identify biomarkers with prognostic application to predict disease outcome and provide innovative laboratorial tools for precise medical applications and therapeutic interventions for managing COVID-19. The general hypothesis is that soluble mediator profiles and shifts in serum and tracheal aspirate samples differs in timing and magnitude in COVID-19 patients according to disease outcome. To test this proposal, the levels of chemokines, pro-inflammatory/regulatory cytokines, and growth factors were quantified in serum and tracheal aspirates from critically ill COVID-19 patients at distinct time points (D0, D2-6, D7, D8-13 and D>14-36) upon Intensive Care Unit (ICU) admission. Our data demonstrated that a massive inflammatory storm of soluble mediators was observed in COVID-19 patients with augmented levels observed serum samples from COVID-19 patients. Moreover, a more pronounced increase was observed in patients who progress to death as compared to those evolving to discharge. Conversely a more restricted up-regulation of soluble mediators were observed in tracheal aspirate samples, indicating that systemic and local compartments diverge in their inflammatory/regulatory milieu.

A massive pro-inflammatory profile mediated by IL-1β, IL-6, TNF and IFN-γ together with lower levels of IL-10 was a systemic hallmark of critically ill COVID-19 patients. On the other hand, a mild pro-inflammatory profile exclusively mediated by increased IL-1β, with unaltered levels of IL-6 and TNF and lower levels of IFN-γ in IL-10-regulated microenvironment, was highlighted in the airway compartment. The analysis of soluble mediators according to disease outcome, demonstrated a further up-regulation of systemic soluble mediators in patients progressing to death. Conversely, in the tracheal aspirate, lower levels of soluble mediators were found in patients progressing to death. The analysis of magnitude of changes of soluble mediators further corroborates the differences between systemic and airway compartmentalized immune response. This phenomenon was consistently observed along the patient’s follow-up.

Particularly, the genes encoding the cytokines CXCL8 (IL-8) IL-1β and IL-6 show markedly high expression upon SARS-CoV-2 infections (24). In agreement with our findings, previous studies evaluating individual serum samples from COVID-19 patients revealed an enhanced and generalized inflammation, featured by a significant increase in circulating IL-6, IL1RA, CXCL8 levels (25).

Significant elevation of CXCL8, which is a classic neutrophil activation and chemoattractant mediator indicates the presence of these polymorphonuclear cells as pivotal driver of the immunopathogenesis in COVID-19 (14, 25–29). In line with this hypothesis, neutrophils have been found as important conspirators of morbid thrombotic events and severe COVID-19 by the formation of neutrophil extracellular traps (NETs) (28). In fact, NETs in COVID-19 are mediated directly by SARS-CoV-2 (29). Although all these preliminary studies were performed with a limited number of patient samples, our findings agree with these reports, demonstrating robust production of the neutrophil-chemoattractant chemokine CXCL8 as a universal COVID-19 mediator in systemic and airway compartments. Thus, the present and previous finds are indicative that CXCL8-neutrophil axis would be a putative target for therapeutic interventions (26).

Regarding the proinflammatory cytokine IL-1β, data reports show controversial results with either not significant differences (25) or increased levels in COVID-19 patients (30). Our findings indicated elevated levels of IL-1β only systemically in COVID-19 patients and no significance was found either in airway or when groups with distinct outcomes were compared. The divergencies observed between previous studies and ours may be due to the lower sample size in previous studies and age and gender biases, which were levelled in our study. However, the role of IL-1β may not be dismissed or disregarded. Significantly elevated levels of IL-1β modulator, IL-1RA, were found in COVID-19 patients in serum and tracheal aspirate samples, which could partially explain the lower levels of IL-1β in airway compartment. IL-1RA was strikingly increased in previous studies (25), bringing about a potential universal biomarker for COVID-19 follow-up. In addition to participating to the inflammasome cascade and proinflammatory mechanisms, it has been proposed that IL-1β triggers augmented expression of the SARS-CoV-2 co-receptor TMPRSS2, facilitating virus cell entry through mechanisms involving the p38 MAPK-GATA2 pathways (31). Therefore, additional studies in humans and mouse models are still needed to unravel the role of IL-1β during severe COVID-19.

Our findings provide evidence that while a massive storm is elicited systemically, deficiencies and gaps in the local immune response are in fact associated with poor prognosis in critically ill COVID-19 patients. IL-10-mediated regulatory bias at airway compartment, with restricted pro-inflammatory milieu may orchestrate these gaps and deficient leading ultimately to fatal outcome. In agreement with our findings, for SARS-CoV-2, severe immunosuppression was found as a feature of COVID-19 patients, placing regulatory mechanisms in the spotlight for understanding disease severity in COVID-19 (23). In fact, our findings show that IL-10 presented unique and coordinative kinetics timelines in serum and tracheal aspirate samples. This regulatory cytokine presented a bimodal distribution with peaks at D2-6 and D8-13 and a drop at D7 for both tracheal aspirates and serum samples. IL-10 was the sole regulatory cytokine with similar kinetics in serum and tracheal aspirate samples, indicating full communication between the systemic and airway compartments.

In view of the role of IL-6 in triggering the associated pathology, several therapeutic proposals have been evaluated to block the pathways of induction of this cytokine. Preliminary studies indicate that Tocilizumab (a recombinant humanized monoclonal antibody) would be able to immediately improve the clinical outcome in critically ill patients with COVID-19, due to the blockage of the febrile and inflammatory response triggered by IL-6, being an effective treatment for reducing mortality, reducing need for mechanical and noninvasive ventilation (32, 33). Most patients under IL-6 inhibition show clinical improvement of symptoms such as hypoxia and changes in computed tomography (CT) opacity immediately after treatment, suggesting that this could be an efficient therapy for the treatment of COVID-19 with decreased 28-day all-cause mortality (33).

Recently, an investigation with a large cohort of patients proposed that serum IL-6 and TNF levels should be considered in the management and treatment of patients with COVID-19 to stratify prospective clinical trials, guide resource allocation and inform therapeutic opportunities (17). In the present study, IL-6 and TNF levels were significantly elevated in serum, but not in airways, suggesting that the highly regulated airway microenvironment may control these two inflammatory mediators. Therefore, although promising, these two mediators should be considered carefully as targets for COVID-19 therapy with anti-IL-6 and anti-TNF monoclonal antibodies.

Regarding the Interferon pathway, specific polymorphisms in genes related to cytokines are strongly associated with disease outcome, such as Interferon related genes (34, 35). Recent investigations have shown that a worst COVID-19 outcome is closely associated to polymorphisms in genes related to the production of proinflammatory IFN-γ and IL-12 (25). In fact, our results show lower levels of IFN-γ in the airway of critically ill COVID-19 patients that progress to death, which suggests the protective role of this cytokine against SARS-CoV-2 infection. In addition, high expression of IFNAR2 interferon receptor subunit reduced the chances of severe COVID-19, indicating that the proinflammatory profile of cytokines are important to disease outcome in COVID-19 (34, 35). In fact, lower levels of circulating IFN-γ were associated to a higher risk of lung fibrosis in COVID-19 patients (36). IFN-γ is a key proinflammatory cytokine that plays a role in improving antigen presentation and inducing of enzymes controlling viral replication. IFN-γ is secreted primarily by activated lymphocytes and, in the context of viral infection or inflammation, IFN-γ modulates not only the transcription of the MHC class I genes in antigen presenting cells, but also multiple components of the antigen-processing and peptide-loading pathways (37). IFN-γ enhances the generation of peptides and immunoproteasome, which is essential for mounting highly specific adaptive responses (37). IFN-γ is also a key cytokine that expands and activates macrophage function present in lungs from severe COVID-19 patients (38).

Although severe COVID-19 immunological profile was initially investigated, a timeline of immune events was not specifically drawn, and it remains to be known the best timing for cytokine attack using specific treatments. In the present investigation, two kinetic patterns of soluble mediators were observed in COVID patients. While a bimodal distribution is observed in the serum of COVID patients, a unimodal peak around D7 is observed in most soluble biomarkers in tracheal aspirate samples from critically ill patients, corroborating the divergent cytokine storm in the systemic and airway compartments. Systems biology tools further demonstrated that COVID-19 display distinct eccentric soluble mediator networks as compared to controls, with opposite profiles observed in serum and tracheal aspirates. Regardless the systemic or compartmentalized microenvironment, networks from patients progressing to death were linked to a pro-inflammatory/growth factor-rich, highly integrated center. Conversely, patients evolving to discharge exhibited networks of weak central architecture, with lower number of neighborhood connections and clusters of pro-inflammatory cytokines and regulatory molecules.

The present study has some limitations. The impact of comorbidities and their association with the levels of serum and airway soluble mediators were not performed in this present investigation. Additional analyses are still required to address this issue. Another limitation that should be disclosed is the fact that critically ill COVID-19 patients were either admitted directly to ICU and intubated immediately or first hospitalized in the regular COVID ward and then transferred to the ICU, and the time points of sample collection were considered only upon ICU admission.

All in all, this investigation with robust sample size landed a comprehensive snapshot of the systemic and local divergencies that clearly showed the distinct immune responses driven by SARS-CoV-2 early on in critically ill COVID-19 patients.

## Data Availability Statement

The original contributions presented in the study are included in the article/**Supplementary Material**. Further inquiries can be directed to the corresponding authors.

## Ethics Statement

The studies involving human participants were reviewed and approved by the Ethical Committee of Instituto René Rachou/FIOCRUZ-Minas (CAAE: 42560721.7.0000.5091) and Hospital das Clínicas da Faculdade de Medicina de Ribeirão Preto - Universidade de São Paulo/USP-RP (CAAE: 30816620.0.0000.5440). The patients/participants provided their written informed consent to participate in this study.

## Author Contributions

Designing research study: VB, CB, MM, MA-M, JC-d-R, and OM-F. Funding Acquisition: FG, AT-C, JC-d-R, and OM-F. Conducting experiments: JG, ÁLR, GF, AAL, and AC-A. Sample collection and handling: CM, AAL, TCF-S, FM, VS, IA, AP, LF, SP, DF, and AM. Medical and Advisory Committee: MAM, AT-C, FG, VB, and CB. Acquiring Medical Records: PM, LA, DP, HR, DF, and MM. Analyzing data: JG, JB-d-S, VP-M, MA, AC-A, JC-d-R, and OM-F. Writing the manuscript: JG, JC-d-R, and OM-F. Revising the manuscript: All authors. All authors contributed to the article and approved the submitted version.

## Funding

This study was funded by Fundação de Amparo à Pesquisa do Estado de Minas Gerais (FAPEMIG), grant # APQ-00432-20 and APQ-01499-21. The study was also supported by the Conselho Nacional de Desenvolvimento Científico e Tecnológico – CNPq and Fundação de Amparo à Pesquisa do Estado de São Paulo (FAPESP). The study was carried out by students enrolled at the Programa de Pós-Graduação em Ciências da Saúde (FIOCRUZ-Minas) and Programa de Pós-Graduação em Microbiologia (UFMG), supported by the Coordenação de Aperfeiçoamento de Pessoal de Nível Superior (CAPES).

## Conflict of Interest

The authors declare that the research was conducted in the absence of any commercial or financial relationships that could be construed as a potential conflict of interest.

## Publisher’s Note

All claims expressed in this article are solely those of the authors and do not necessarily represent those of their affiliated organizations, or those of the publisher, the editors and the reviewers. Any product that may be evaluated in this article, or claim that may be made by its manufacturer, is not guaranteed or endorsed by the publisher.
