# Supplementary material for: Timeline Kinetics of Systemic and Airway Immune Mediator Storm for Comprehensive Analysis of Disease Outcome in Critically Ill COVID-19 Patients
Source: Front Immunol. 2022 Jun 3;13:903903. doi: 10.3389/fimmu.2022.903903 (PMC9204232; doi:10.3389/fimmu.2022.903903)
Supplement: Supplementary file 1 [file Image_1.pdf]

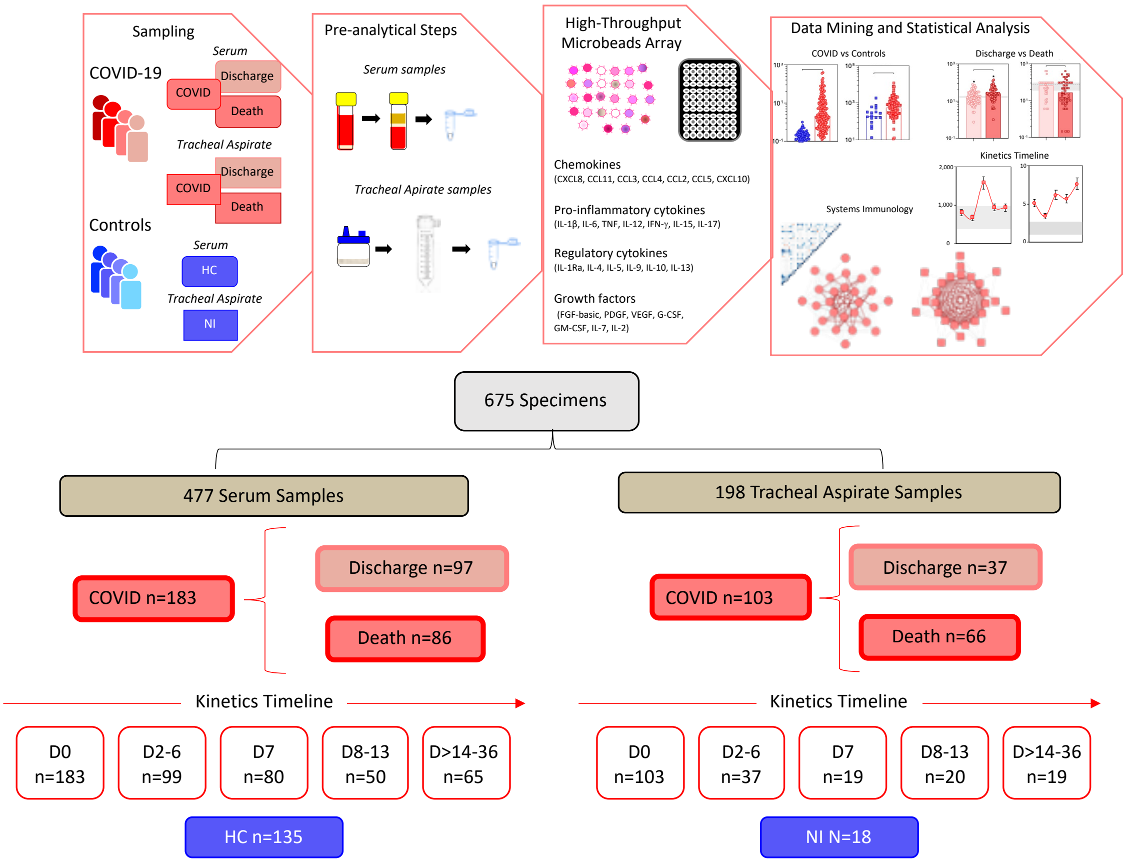

Supplementary Figure 1 – *Compendium of the study population and methods.* An overview of sampling, pre-analytical steps, high-throughput analysis of soluble immune mediators, data mining and statistical analysis are provided in the top panels. This investigation comprised a cross-sectional study including 675 human specimens (477 serum samples and 198 tracheal aspirate samples) from critically ill COVID-19 patients ("COVID"), serum samples from pre-pandemic healthy controls (HC) and tracheal aspirates from non-infected patients (NI) under mechanical ventilation at ICU. For assessing the systemic immune response, the "COVID" group comprised 477 serum samples from 183 COVID-19 patients (72 females and 111 males – age range of 18-90 years-old, median age of 65 years-old), obtained at five consecutive time points (Days = D), including: D0 (n=183), D2-6 (n=99), D7 (n=80), D8-13 (n=50) and D>14-36 (n=65) after ICU admission. "COVID" group was further categorized according to disease outcome: discharge (n=97) or death (n=86). The control group was composed of 135 serum samples from age-matched pre-pandemic healthy controls referred as: "HC" group. For assessing the airway compartmentalized immune response, the "COVID" group was composed of 198 tracheal aspirate (TA) samples from 103 COVID-19 patients (39 females and 64 males – age range of 18-90 years-old, median age 64 years-old), collected

at five consecutive time points (Days = D), including: D0 (n=103), D2-6 (n=37), D7 (n=19), D8-13 (n=20) and D>14 (n=19) after ICU admission. The “COVID” group was further categorized according to the disease outcome, referred as: “Discharge” (n=37) or “Death” (n=66). The control group was composed of 18 non-infected patients under mechanic ventilation due to trauma or burning, all presenting negative diagnosis of SARS-CoV-2 infection by RT-PCR, referred as “NI” group.
