# Supplementary material for: Timeline Kinetics of Systemic and Airway Immune Mediator Storm for Comprehensive Analysis of Disease Outcome in Critically Ill COVID-19 Patients
Source: Front Immunol. 2022 Jun 3;13:903903. doi: 10.3389/fimmu.2022.903903 (PMC9204232; doi:10.3389/fimmu.2022.903903)
Supplement: Supplementary file 2 [file Image_2.pdf]

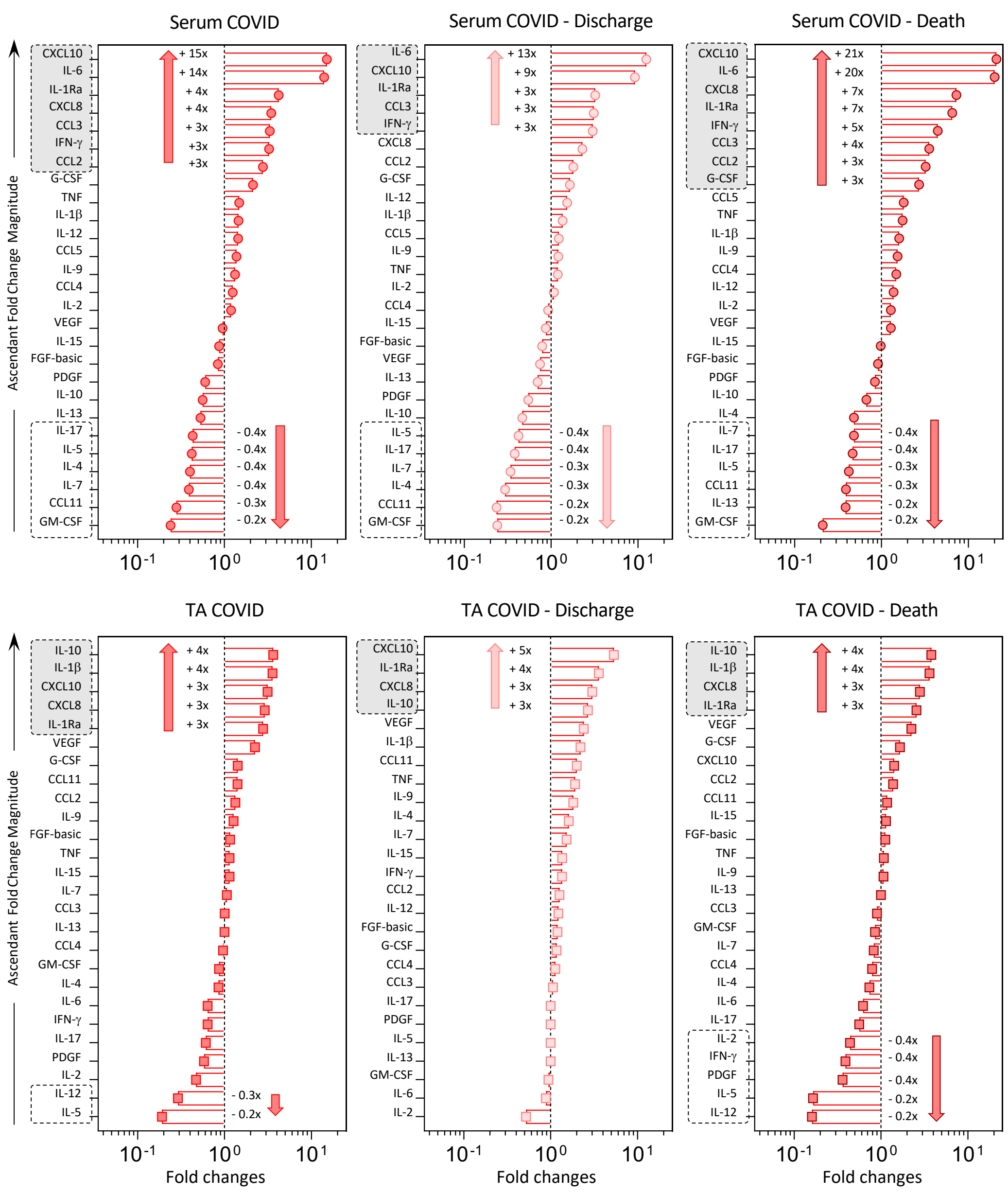

Supplementary Figure 2 – *Magnitude of changes in serum and airway soluble immune mediators from critically ill COVID-19 patients*. The magnitude of changes in the levels of chemokines (CXCL8, CCL11, CCL3, CCL4, CCL2, CXCL10), pro-inflammatory cytokines (IL-1β, IL-6, TNF, IL-12, IFN-γ, IL-15, IL-17), regulatory cytokines (IL-1Ra, IL-4, IL-5, IL-9, IL-10, IL-13) and growth factors (FGF-basic, PDGF, VEGF, G-CSF, GM-CSF, IL-7 and IL-2) were calculated for individual samples from COVID-19 patients – "COVID" (serum = ●, n=183; TA = ■, n=103), further classified according to disease outcome: referred as "Discharge" (serum = ○, n=97; TA = □; n=37) or "Death" (serum = ●, n=86; TA = ■, n=66). Measurements of soluble mediators were carried by Luminex Bio-plex platform as described in material and methods section. The magnitude of

changes in serum soluble and TA immune mediators was calculated as the proportion ratio of individual values according to the median values observed in serum samples from pre-pandemic healthy controls (HC) and tracheal aspirates from non-infected patients (NI). The results are expressed as median values and presented in bar chart format as ascendant fold change magnitude. The soluble mediators with decreased levels (<-0.4) and increased levels (>+3) were underscored by upward or downward arrows, respectively.
