# Supplementary material for: Timeline Kinetics of Systemic and Airway Immune Mediator Storm for Comprehensive Analysis of Disease Outcome in Critically Ill COVID-19 Patients
Source: Front Immunol. 2022 Jun 3;13:903903. doi: 10.3389/fimmu.2022.903903 (PMC9204232; doi:10.3389/fimmu.2022.903903)
Supplement: Supplementary file 3 [file Image_3.pdf]

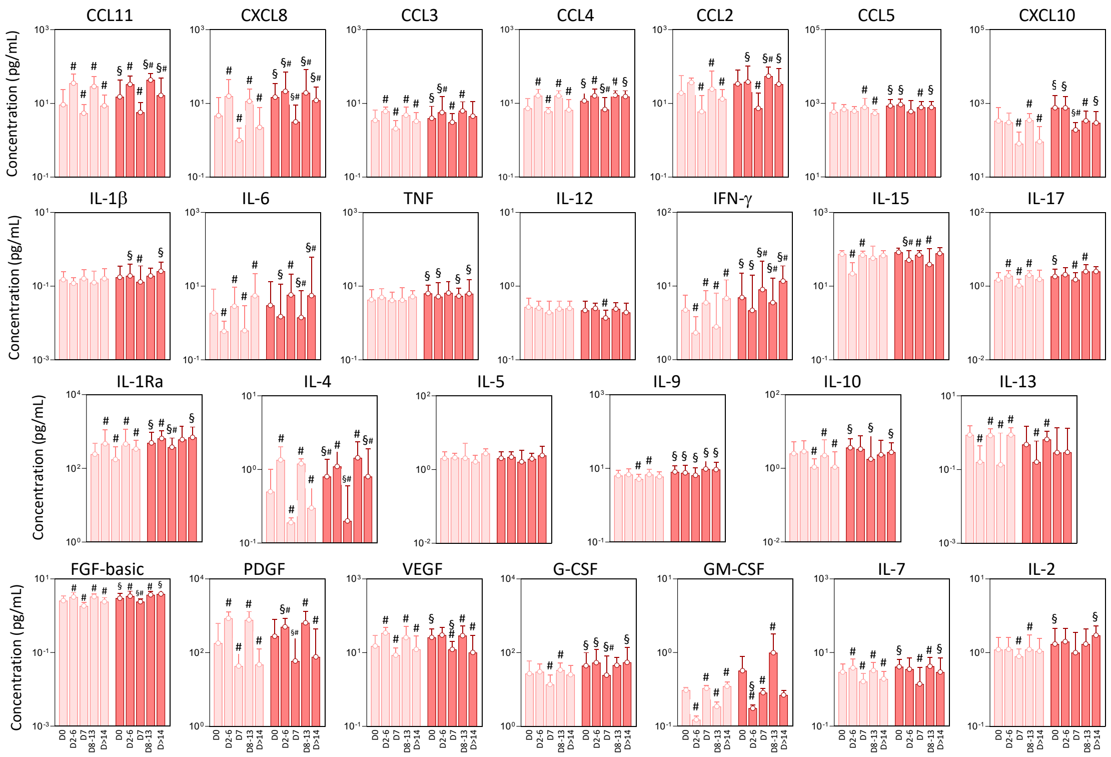

Supplementary Figure 3 – *Kinetic timeline of serum soluble immune mediators in critically ill COVID-19 patients, according to disease outcome.* The kinetic profile of chemokines (CXCL8, CCL11, CCL3, CCL4, CCL2, CCL5, CXCL10), pro-inflammatory cytokines (IL-1β, IL-6, TNF, IL-12, IFN-γ, IL-15, IL-17), regulatory cytokines (IL-1Ra, IL-4, IL-5, IL-9, IL-10, IL-13) and growth factors (FGF-basic, PDGF, VEGF, G-CSF, GM-CSF, IL-7 and IL-2) was evaluated in serum samples from critically ill COVID-19 patients (n=183), further categorized according to disease outcome, referred as "Discharge" (, n=97) or "Death" (, n=86). The kinetic timeline was evaluated by cross-sectional analysis at four consecutive time points (Days = D), including: D0 (n=97;86), D2-6 (n=36;63), D7 (n=54;26), D8-13 (n=20;30), and D>14-36 (n=43;22) after ICU admission. Measurements were carried out by high-throughput microbeads array as described in Material and Methods section. The results are presented as bar chart of median values and interquartile range at each time point along the kinetic timeline. Significant differences at $p<0.05$ are identified by hashtag (#) for comparisons with immediately preceding time-point and by "§" for comparisons between "Discharge" *vs* "Death" subgroups at matching time-points.
